# Comparative Analysis of Satellite DNA in *Dasypyrum* Species: Identification of Chromosomal Markers for V and V^b^ Subgenomes

**DOI:** 10.3390/plants14243819

**Published:** 2025-12-15

**Authors:** Anna I. Yurkina, Viktoria M. Sokolova, Ekaterina D. Badaeva, Daniil S. Ulyanov, Gennady I. Karlov, Mikhail G. Divashuk, Pavel Yu. Kroupin

**Affiliations:** 1 All-Russia Research Institute of Agricultural Biotechnology, Timiryazevskaya St. 42, 127434 Moscow, Russia; 2 N.I. Vavilov Institute of General Genetics, Russian Academy of Sciences, Gubkin St. 3, 119991 Moscow, Russia

**Keywords:** *Dasypyrum*, fluorescence in situ hybridization, V genome, V^b^ genome, satellite repeats

## Abstract

The genus *Dasypyrum* represents a valuable source of beneficial traits for wheat improvement, yet the cytogenetic organization of its genomes, particularly of the satellite repeats, remains poorly understood. This study aimed through comparative analysis of satellite DNA in diploid *D. villosum* (W6 21717, V genome) and tetraploid *D. breviaristatum* (PI 516547, VV^b^ genomes) to reveal the evolutionary dynamics of their subgenomes and to identify species-specific chromosomal markers. We performed whole-genome sequencing, bioinformatic analysis, and fluorescence in situ hybridization (FISH). Bioinformatic screening identified 14 satellite repeats in the *D. breviaristatum* genome (CL9, CL95, CL100, CL110, CL127, CL133, CL134, CL135, CL147, CL153, CL165, CL169, CL173, and CL197), which were classified by copy number: one as high-copy (CL9, ≥0.6%) and the rest as low-copy (<0.29%). Their monomer sizes ranged broadly from 118 to 1118 base pairs. Most repeats showed varying degrees of homology with known sequences from the Triticeae family, and one repeat, CL165, had no detectable homologs in existing databases. FISH analysis subdivided repeats into three groups: predominantly terminal (CL100, CL110, CL134, CL135, CL147, CL165, CL169, CL173, and CL197), pericentromeric (CL127 and CL133), and mixed localization (CL9). Significant species-specific differences were revealed, including emergence of tetraploid-specific repeats (CL110, CL134, CL135, CL147, CL165, and CL173) and the reorganization of conserved sequence distribution. Notably, the repeat CL135 was identified as a specific marker for the V subgenome within the allopolyploid *D. breviaristatum*. The obtained data support the allopolyploid origin of *D. breviaristatum* and demonstrate that these two species are genetically distinct but evolutionarily closely related. Chromosomal markers developed based on newly discovered satellite repeats open new avenues for investigating genomic architecture and evolutionary relationships within the genus *Dasypyrum*, as well as for identifying its chromatin in distant hybrids.

## 1. Introduction

The genus *Dasypyrum* (syn. *Haynaldia*), being a wild relative of wheat, includes two species: the annual *Dasypyrum villosum* (2n = 2x = 14) and the perennial *D. breviaristatum* (2n = 2x = 14 and 2n = 4x = 28) [1,2,3,4]. Due to significant genomic diversification, the genomes of *D. villosum* and diploid *D. breviaristatum* were designated with the symbols V and V^b^, respectively [5]. For a long time, the genomic structure of tetraploid *D. breviaristatum* remained incompletely understood; in particular, it was not clear whether it was an auto- or allopolyploid [1,6,7,8]. However, subsequent studies on the genomic organization of *Dasypyrum* showed that tetraploid *D. breviaristatum* has an allopolyploid structure—VV^b^ [9]. This conclusion was supported by phylogenetic analysis, which revealed significant divergence between *D. villosum* and *D. breviaristatum* despite their close relationship [5].

Interest in this genus is driven by the fact that cultivated wheat (*Triticum aestivum* L.), being one of the world’s most important cereal crops, faces threats from the spread of diseases and climate change [10]. Long-term selective breeding has led to a narrowing of its genetic diversity. Because of this, wild relatives of particular *Dasypyrum* species are of particular value as sources of new beneficial genes for expanding the wheat gene pool [11,12]. *Dasypyrum* species possess valuable agronomic traits such as disease resistance, high protein content, and drought tolerance, which can significantly enrich the genetic diversity of the wheat gene pool for breeding purposes [13,14].

A classic example of successful introgression of resistance genes from *D. villosum* into wheat is the development of the translocational line T6VS·6AL, which carries the *Pm21* gene [15,16]. This gene provides a high level of resistance to powdery mildew caused by the pathogen *Blumeria graminis* f. sp. *tritici* (Bgt) and has been widely used in Chinese wheat breeding programs [17,18]. The T4DL·4V#6S and T7DL·7DS-4V#6L translocation lines with the *Pm4VL* gene are also known [19]. In addition, the genetic potential of *D. villosum* is not limited to chromosome 6V, as the chromosome 5V also contains valuable resistance genes such as *Pm55* and *Pm5V* [20].

Stem rust, caused by *Puccinia graminis* f. sp. *tritici*, is one of the most destructive wheat diseases. The emergence of the Ug99 (TTKSK) race and its derivatives has led to the loss of effectiveness of many resistance genes, such as *Sr24*, *Sr31*, and *Sr38* [21,22,23]. However, *D. villosum* exhibits resistance to Ug99, conferred by the *Sr52* gene located on the long arm of chromosome 6V [24,25] and the SrTA10276-2V gene on chromosome 2V [26].

Stripe rust resistance genes have been identified on *D. villosum* chromosomes 1V, 3V (*YrCD-3*), 5V (*Yr5V*), and 7V (*Yr7VS*) [27,28,29,30]. The common wheat–*D. breviaristatum* 2V^b^(2D) substitution line D11-5 was reported to exhibit high resistance to this disease and good agronomical traits [31]. In addition, *Dasypyrum* species showed resistance to wheat spindle streak mosaic virus (*Wss1*) [32], eyespot (*Pch3* = *PchDV*) [33], cereal cyst nematodes (*CreV*) [34], Fusarium head blight [35], and leaf rust [36]. They also exhibit tolerance to salt, drought, and heat stressors [37,38,39,40], confirming their value as a source of traits for wheat breeding.

FISH (fluorescence in situ hybridization) techniques, utilizing both universal and species-specific probes, are widely used for the precise identification of chromosomes and the analysis of their structural rearrangements [41,42,43,44,45,46]. Detailed karyotyping of *Dasypyrum* species provides a foundation for subsequent comparative and evolutionary studies within the genus and with other members of the tribe Triticeae.

Analyses of repetitive DNA sequences, particularly satellite repeats, are an important direction in cytogenetic research of *Dasypyrum*, because they are used as markers for chromosome identification and serve as indicators of evolutionary changes and chromosomal rearrangements. Repeats constitute a significant portion of plant genomes and reflect both the degree of relatedness between species and internal organization of their chromosomes [47,48,49,50,51]. FISH techniques enable the visualization of these repeats, allowing comparison of their distribution in closely related species. The similarity or difference of chromosomal arrangement of the same satellite sequences on chromosomes of related species provides valuable insights into the degree of relatedness of their subgenomes and the evolutionary processes that have shaped their genetic architecture [52,53,54]. Therefore, analysis of repeat distribution using FISH could be a key tool for understanding phylogenetic relationships and genome divergence within the genus *Dasypyrum*.

The aim of this study is to conduct a comparative cytogenetic analysis of satellite repeats in diploid *D. villosum* and tetraploid *D. breviaristatum* to reveal the evolutionary dynamics of their subgenomes and develop species-specific chromosomal markers. To achieve these goals, it is necessary to: (1) identify and characterize a set of satellite repeats in the *D. breviaristatum* genome using whole-genome sequencing and bioinformatic analysis; (2) perform detailed cytomolecular karyotyping by mapping the chromosomal distribution of these repeats in both species using in situ fluorescent hybridization (FISH); (3) to conduct a comparative analysis of the FISH structure in order to identify conservative and species-specific organizational features.

## 2. Results

### 2.1. Satellite Repeats Characterization

Based on the whole-genome sequencing data, we identified fourteen repeats in *D. breviaristatum*: ten high-probability satellite repeats (CL9, CL95, CL110, CL127, CL134, CL135, CL147, CL165, CL169, and CL197) and four low-probability satellite repeats (CL100, CL133, CL153, and CL173). Characteristics of the identified repeats are provided in Table S1, including repeat monomer consensus sequences, TAREAN graphs, repeat copy numbers in the genome, homology with published sequences in the NCBI database, and sequences from other studies.

Based on bioinformatics analysis, all repeats were classified as low-copy (<0.29%), medium-copy (0.3–0.59%), and high-copy (≥0.6%). CL9 was identified as the only high-copy repeat, while all other repeats were low-copy.

Additionally, the monomer sizes of all satellite repeats were determined (Table S1). High-confidence satellite repeats are represented by sequences ranging from 178 to 863 base pairs (bps): CL9 (337 bps), CL95 (358 bps), CL110 (570 bps), CL127 (863 bps), CL134 (568 bps), CL135 (380 bps), CL147 (376 bps), CL165 (842 bps), CL169 (178 bps), and CL197 (566 bps). Low-confidence repeats have the following sizes: CL100 (118 bps), CL133 (504 bps), CL153 (1118 bps), and CL173 (118 bps). Thus, the overall range of monomer sizes for all identified satellites varies from 118 to 1118 bps.

Comparison of the nucleotide sequences of the newly discovered repeats with those previously published in the NCBI database revealed that only CL165 showed no homologs. For the remaining repeats, the percentage identity with known similar sequences ranged from 65% to 98%, indicating that they diverged from previously reported sequences.

Assessments of the homologies of the repeats we identified in *D. breviaristatum* revealed that most sequences shared significant similarity with known repetitive elements in cereal crops. These satellite repeats have been found in a wide range of species, including *Thinopyrum bessarabicum* (CL97, CL157, CL192), *Agropyron cristatum* (ACRI_TR_CL20, ACRI_TR_CL85), *Aegilops crassa* (CL244, CL261), and *Elymus* (StLIB96, StLIB117). Three of the repeats we identified (CL95, CL135, and CL147) showed homology to the satellite repeat ACRI_TR_CL20 from *A. cristatum* (68–90%). Repeats CL9, CL134, and CL100 demonstrated homology with the following FISH-positive sequences from wheat, pTa-173, pTa-1, pTa-505, and pTa-835, respectively (86–96%). Three repeats shared similarity with spelt-like sequences: CL135 and CL147 with Spelt52.2 from *Ae. speltoides* (68% and 93%) and CL169 with Tri-MS-6 from *T. aestivum* (75%). Repeats CL9, CL95, and CL100 exhibited similarity with retrotransposons from barley and rye (88–93%). Furthermore, repeats CL135 and CL147 showed similarity with the rye tandem repeat pSc200 (70–75%), while repeat CL110 showed similarity with pSc250 (78%).

In addition, we assessed similarity between the repeats we identified and oligoprobe sequences reported in various studies (Table S1). This analysis revealed that repeat CL9 showed similarity to the sequences of pAs1, a member of AFA-family (85–95%), as well as to oligo-pTa535 (86%). Repeats CL133 and CL169 were similar to sequences from *Th. elongatum*: CL133 with oligo-7E-430 (98%) and CL169 with oligo-7E-744 (97%). Repeat CL173 shared a 91% similarity with oligo-6H-2-100 from *Th. intermedium*. A number of repeats were similar with oligos developed for *T. aestivum* (Chinese Spring), specifically CL133 with oligo-5D151 (98%), CL135 with oligo-Td25 (85%), and CL147 with oligo-Td25 and DP4J27982 (98% and 100%).

### 2.2. Mapping of Satellite Repeats on D. villosum and D. breviaristatum Chromosomes

**Figure 1:**
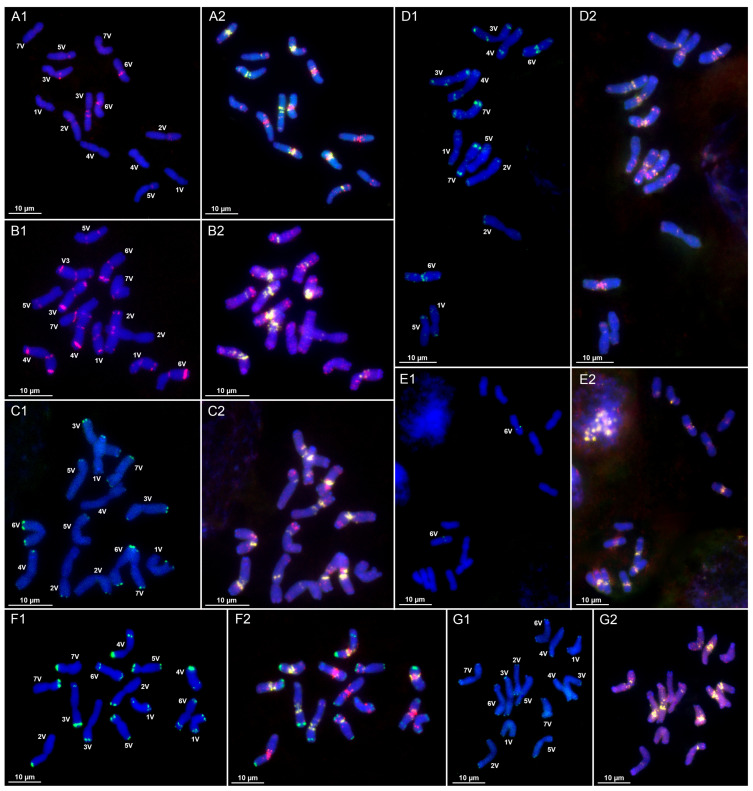
Chromosomal localization of satellite repeats on metaphase cells of *D. villosum* (**A1**–**G1**): (**A1**)—CL133 (red) + CL134 (green), (**B1**)—CL95 (red) + CL147 (green), (**C1**)—CL135 (red) + CL197 (green), (**D1**)—CL110 (red) + CL9 (green), (**E1**)—CL153 (red) + CL127 (green), (**F1**)—CL165 (red) + CL100 (green), and (**G1**)—CL173 (red) + CL169 (green); (**A2**–**G2**)–pSc119 FAM (green) + pAs1 TAMRA (red) + (GAA)_10_ (yellow). The bar indicates 10 µm.

**Figure 2:**
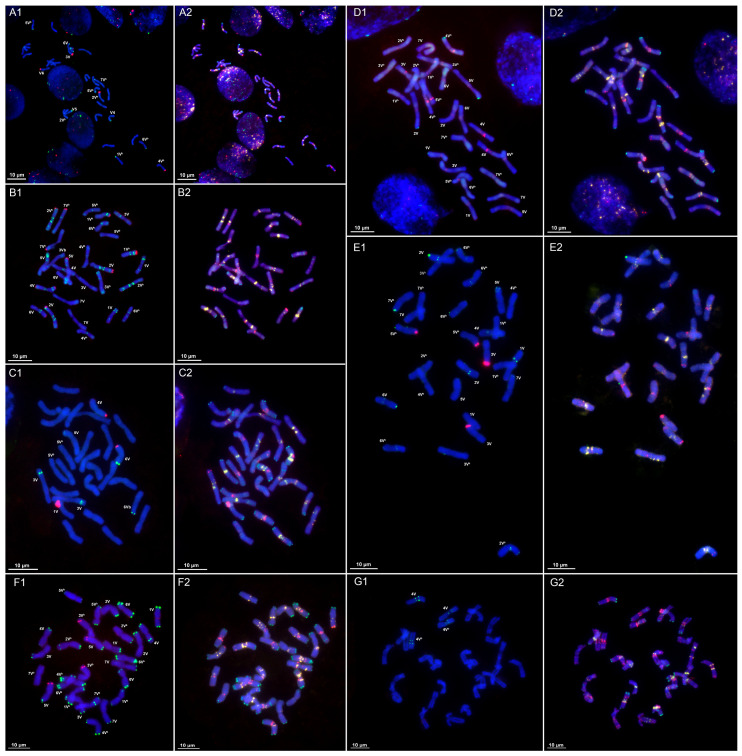
Chromosomal localization of satellite repeats on metaphase cells of *D. breviaristatum* (**A1**–**G1**): (**A1**)—CL95 (red) + CL147 (green), (**B1**)—CL110 (red) + CL9 (green), (**C1**)—CL135 (red) + CL197 (green), (**D1**)—CL133 (red) + CL134 (green), (**E1**)—CL173 (red) + CL169 (green), (**F1**)—CL165 (red) + CL100 (green), and (**G1**)—CL153 (red) + CL127 (green); (**A2**–**G2**)—pSc119 FAM (green) + pTa-535 TAMRA (red) + (GAA)_10_ (yellow). The bar indicates 10 µm.

CL9: The satellite repeat CL9 occurs on all chromosome pairs of *D. villosum* (Figure 1D1,D2 and Figure S1). It forms terminal signals on the short arm chromosome 1V, where minor signals are also observed; chromosome 2V shows subterminal signals on both arms; chromosome 3V possesses subterminal signals on both arms with an additional interstitial signal on the long arm; chromosome 4V displays distal CL9 signals on the short arm and weak subterminal signals on the long arm; chromosome 5V shows minor signals; chromosome 6V exhibits intense pericentromeric signals along with minor signals on both arms; and chromosome 7V demonstrates the most intense subterminal signals on both long and short arms. In *D. breviaristatum*, CL9 is found on all chromosomes of both V and V^b^ subgenomes (Figure 2B1,B2 and Figure S2) and displays multiple signals with predominantly proximal and distal localization.

CL95: In *D. villosum*, CL95 is localized pericentromerically and subterminally on both arms of chromosomes 1V, 3V, and 4V; pericentromerically on 7V; distally on 5VL; and pericentromerically and subterminally on the long arm of 6VL. Chromosome 2V shows that the heteromorphic pattern CL95 is localized subterminally on the long arm of one homolog, while in the second we see a subterminal signal on the short arm and a weak subterminal signal on the long arm (Figure 1B1,B2 and Figure S1). In *D. breviaristatum*, CL95 exhibits terminal localization—on both arms of chromosome 3V, and on 6VL, 2V^b^L, and 4V^b^L (Figure 2A1,A2 and Figure S2).

CL100 forms subterminal and terminal clusters on *D. villosum* chromosomes (Figure 1F1,F2 and Figure S1). Terminal signals are present on 2VS, 3VS, and 7VS arms; chromosomes 1V and 6V display terminal signals on both arms, with an additional subterminal signal in the long arm; CL100 localizes terminally on both arms of chromosome 5V; and one homolog of 4V has a signal at the end of short arm, while another homolog exhibits intense distal signals on the long arm. In *D. breviaristatum*, CL100 demonstrates a predominantly terminal location either on both arms (1V, 3V, 4V, 6V, 1V^b^, 4V^b^, 5V^b^, and 6V^b^), on the short arm only (5V, 7V, and 7V^b^), or on the long arm only (2V and 2V^b^) (Figure 2F1,F2 and Figure S2). No signal was detected on 3V^b^. It should also be noted that several chromosomes are heteromorphic in signal distribution: one homolog of 3V lacks a terminal signal on the short arm; chromosome 4V displays a distal signal on the short arm with the least intense terminal signal; chromosome 2V^b^ has a distal signal on the short arm; chromosome 4V^b^ shows a subterminal signal for CL100; and no CL100 signals were detected on one homolog of chromosome 7V^b^.

CL110 is not detected by FISH in *D. villosum* (Figure 1D1,D2). In *D. breviaristatum*, it is located predominantly in terminal chromosome regions (Figure 2B1,B2 and Figure S2)—on both homologs of 2VS, 1V^b^S, and 7V^b^S, and on only one homolog of each of 4V, 5VS, 6VL, and 2V^b^S.

CL127 is distributed proximally on 6VS in *D. villosum* (Figure 1E1,E2 and Figure S1) and on both arms of 4V and 4V^b^ in *D. breviaristatum* (Figure 2G1,G2 and Figure S2).

CL133 produces proximal signals on chromosomes 2VS, 3VL, 5VS, and 6VL of *D. villosum*, with one homolog of 3V showing an additional pericentromeric signal (Figure 1A1,A2 and Figure S1). In *D. breviaristatum*, CL133 also exhibits proximal localization on all chromosomes, with the most intense signals on 4V, 4V^b^, and 5V^b^ (Figure 2D1,D2 and Figure S2).

CL134: No hybridization signals for CL134 are detected on *D. villosum* chromosomes (Figure 1A1,A2 and Figure S1). In *D. breviaristatum*, CL134 localizes on 1VL, 2VL, 5VS, 6VL, 1V^b^L, 4V^b^S, and 6V^b^S; however, in all these chromosome pairs the signal is present on one homolog only (Figure 2D1,D2 and Figure S2).

CL135: Diploid *D. villosum* lacks this repeat (Figure 1C1,C2), while one homolog of each of the *D. breviaristatum* chromosomes pairs 1VS, 4VS, and 6VL carries terminal CL135 signals, with the most intense signal being on 1VS (Figure 2C1,C2 and Figure S2).

CL147: FISH was unable to detect this repeat on *D. villosum* chromosomes (Figure 1B1,B2). CL147 signals distribution on *D. breviaristatum* chromosomes proves to be heterozygous. Distal signals are observed on one 3VS homolog and terminal signals on one homolog of 4VS, 5VS, 1V^b^S, 2V^b^S, 6V^b^S, and 7V^b^S. A single 6V chromosome displays terminal CL147 signals on both short and long arms, and two homologs differ in signal distribution: terminally on the long or subterminally on the short arm 5V^b^ (Figure 2A1,A2 and Figure S2).

CL153: FISH was unable to detect CL153 in *Dasypyrum* species (Figure 1E1,E2 and Figure 2G1,G2).

CL165 is not found in *D. villosum* (Figure 1F1,F2), while in *D. breviaristatum* it produces subterminal signals on two chromosome pairs—5VL and 3V^b^L (Figure 2F1,F2 and Figure S2).

CL169 forms terminal signals on all chromosomes of *D. villosum* (Figure 1G1,G2 and Figure S1), while in *D. breviaristatum* it exhibits terminal and/or proximal location on 1V, 2V, 4V, 5V, 6V, 7V, 2V^b^, 6V^b^, and 7V^b^ (Figure 2E1,E2 and Figure S2). Chromosomes 1V, 7V, 2V^b^, 6V^b^, and 7V^b^ are heterozygous in CL169’s signal distribution.

CL173 is not detected in *D. villosum* using FISH (Figure 1G1,G2), while intense terminal signals appear on 3VL and 5V^b^L of *D. breviaristatum,* along with weak minor signals at 1VL, and on single homologs of each 7VL and 7V^b^S (Figure 2E1,E2 and Figure S2).

CL197 localizes terminally on both arms of 3V, 6V, and 7V, on the long arm of 1V and 4V, and on the short arm of 2V and 5V of *D. villosum* (Figure 1C1,C2 and Figure S1), with chromosome 4V being heterozygous. In *D. breviaristatum*, subterminal CL197 clusters appears on chromosome 2VS, and terminal clusters on both 5V^b^S homologs and on one homolog of each of 4VS, 5VS, and 6V^b^L (Figure 2C1,C2 and Figure S2). It should also be noted, however, that CL197’s signal size varies in *D. breviaristatum*, with the most intense signals observed on chromosomes 3V, 6V, and 6V^b^.

Based on distribution pattern, all satellite repeats identified in the studied *Dasypyrum* species can be divided into three main groups:

Group 1—repeats with predominantly terminal and distal localization. This category includes repeats CL100, CL110, CL134, CL135, CL147, CL165, CL169, CL173, and CL197.

Group 2—repeats with pericentromeric and proximal localization—CL127 and CL133.

Group 3—repeats with complex hybridization patterns. This group includes CL9, characterized by the presence of various signal types: terminal, subterminal, distal, and pericentromeric.

## 3. Discussion

Satellite repeats are the most variable and rapidly evolving components. They are tandemly organized or dispersed DNA sequences that are widely distributed in plant genomes, particularly in cereals [49,55]. These repeats play a crucial role in chromosome organization, being involved in the formation and stabilization of centromeric and telomeric regions, as well as in maintaining genome integrity [56,57,58]. Due to their high variability and species-specificity, satellite repeats serve as valuable markers for studying evolution, phylogenetic analysis, and chromosome identification.

Employment of satellite repeats as chromosomal markers enables detailed analysis of plant genomes. One of the key methods for their physical mapping is fluorescence in situ hybridization (FISH), which provides high visualization accuracy and allows for studying the distribution of these sequences on chromosomes [59,60,61,62].

Our current results have provided detailed information on the genomic and chromosomal organization of *Dasypyrum* species. FISH analysis using satellite repeats enabled the development of new specific molecular cytogenetic markers for the V and V^b^ genome chromosomes of *Dasypyrum*. Comparative FISH analysis revealed both shared features and species-specific differences in satellite DNA organization between diploid *D. villosum* and tetraploid *D. breviaristatum*.

Of the fourteen analyzed repeats, thirteen (CL9, CL95, CL100, CL110, CL127, CL133, CL134, CL135, CL147, CL165, CL169, CL173, and CL197) showed discrete hybridization patterns on chromosomes of at least one species. The repeats CL110, CL134, CL135, CL147, CL153, CL165, and CL173 were not detected in the *D. villosum* genome, indicating that they are either absent or represented by an extremely low copy number in this species. In contrast, the same repeats (with the exception of CL153) displayed clear FISH patterns in the *D. breviaristatum* chromosome genome, often in a heterozygous state, suggesting their divergence or recent emergence in the allopolyploid’s evolution. It is also noteworthy that such a localization pattern on an odd number of chromosomes is characteristic of cross-pollinating species with heterozygous genomes [63].

The localization of group 1 repeats in telomeric regions aligns with the general trend of satellite DNA accumulation in heterochromatic areas at chromosome ends, which is characteristic of many cereal species. Comparison of sequences of the repeats assigned to this group revealed that nearly all of them share similarity with tandem repeats pSc119.2, pSc200, and pSc250 of *S. cereale* and FISH-positive pTa-like sequences of *T. aestivum*, which are widespread in the tribe Triticeae, including genera *Triticum*, *Aegilops*, *Agropyron*, *Dasypyrum*, *Thinopyrum*, *Pseudoroegneria*, and *Avena*, and are detected in both subtelomeric and interstitial chromosomal regions [64,65,66,67,68,69].

Repeats CL110, CL134, CL135, and CL147 also show similarity to StY repeats, with predominantly terminal localization on St-subgenome chromosomes and distal localization on Y-subgenome chromosomes of *Roegneria grandis* [70].

Similar repeats with terminal locations have also been found in other Triticeae species: BSCL and DP4J27982 in *Th. bessarabicum* J^b^-genome [71,72]; STlib_96, and CL69 in *P. libanotica* St genome [73,74]; oligo-D1D in *T. aestivum* D-subgenome [75] and in *D. breviaristatum* V and V^b^ subgenomes [76]; oligo-Td25 in *D. breviaristatum* V^b^ subgenome [76]; Spelt-1 and Spelt52, *Ae. speltoides* S genome [77]; CL244 in *Ae. crassa* X^cr^ subgenome (also in *Th. bessarabicum* J^b^-genome and common wheat B subgenome) [62]; and oligo-7E-744 and oligo-6H-2-100 in *Th. elongatum* (localized on V^b^ subgenome chromosomes of *D. breviaristatum*) [76].

It is noteworthy that no similar sequences were found for CL165, suggesting its unique origin or significant divergence from other repeats.

Given the broad spectrum of genomes containing terminal repeats, we can hypothesize ancient origin from a common ancestor for the repeats CL100, CL110, CL134, CL135, CL147, CL169, CL173, and CL197 identified in the current study. We can also hypothesize their potential role in stabilizing chromosome ends and maintaining structural integrity of genomes.

Considering group 2, the CL133 shares homology with the FAT-repeat of *T. aestivum*, which primarily hybridizes to proximal and pericentromeric regions of the D chromosomes, as well as to chromosomes of the C, D, N, M, S, X^cr^, and U genomes of various *Aegilops* species, the H genome of *H. chilense*, the XX^1^ genome of *H. geniculatum*, and the R genome of *S. cereale*, and also exhibits intense dispersed signals in proximal regions of St genome chromosomes of *P. spicata* [78,79]. Furthermore, CL133 demonstrates similarity with the microsatellite P631 with pericentromeric distribution on chromosomes of the J^r^, J^v^, and St subgenomes of *Th. intermedium*, the J^b^ genome of *Th. bessarabicum*, the St genome of *P. spicata*, and on *T. aestivum* chromosomes (subgenome affiliation was not determined) [80]. It is also similar to the FISH-positive repetitive sequence pTa-451 of *T. aestivum*; oligo-7E-430 from *Th. elongatum* (pericentromeric localization on chromosome 6V^b^ of *D. breviaristatum*); and oligo-5D151 from *T. aestivum*, which localizes on chromosomes 5V, 6V, 7V, 5V^b^, and 6V^b^ of *D. breviaristatum* [76]. These differences in hybridization patterns may be explained by accumulation of these sequences in the pericentromeric region of a common ancestor of *Triticum*, *Aegilops*, *Thinopyrum*, *Secale,* and *Pseudoroegneria*. The number and distribution of these elements changed during subsequent divergence, leading to different hybridization patterns. Although the listed repeats are homologous to each other, some are dispersed from the pericentromeric region to proximal areas.

Only one homolog of repeat CL127 was identified—it was a satellite sequence ACRI_TR_CL85 from *A. cristatum*. As far as its localization has not been characterized, it can be considered that we have discovered a new proximal repeat.

Furthermore, there is CL9 from group 3, characterized by the occurrence of various signal types (terminal, subterminal, distal, and pericentromeric). The corresponding hybridization pattern is supported by CL9’s homology to the Afa family repeats, pAs1 from *Ae. tauschii* and pTa535 from *T. aestivum* [81], which primarily localize in subtelomeric and interstitial chromosomal regions. These sequences are discovered in a broad range of cereal taxa, including *Triticum*, *Hordeum*, *Secale*, and even certain species of *Leymus* and *Psathyrostachys*, indicating their ancient origin predating the divergence of the Triticeae tribe [82,83]. Interestingly, despite the structural conservation of the flanking regions of these repeats, their central part is highly polymorphic, enabling the use of pAs1-like sequences in cytogenetics for identifying individual chromosomes and genomes [84]. Nevertheless, the localization pattern of CL9 differs from the Afa family, which may indicate divergence of the repeat we discovered from the ancestral form shared by the Afa family.

The repeat CL95 is of particular interest as it did not fit into any of the established groups due to its specific patterns in *D. villosum* and *D. breviaristatum*—exhibiting pericentromeric, distal, and subterminal localization in the diploid species, but exclusively terminal localization in the tetraploid. CL95 showed similarity with the terminal and/or subterminal BSCL5 repeat of *Th. bessarabicum* [72]. Different patterns of CL95 in diploid *D. villosum* and tetraploid *D. breviaristatum* can likely be explained by the reduction or redistribution of repetitive sequences, particularly from pericentromeric and interstitial regions, which often occurs during polyploidization and is accompanied by their subsequent accumulation in terminal heterochromatic blocks [85]. The similarity of CL95 with the terminal repeat BSCL5 of *Th. bessarabicum* suggests possible conservation of its telomere-associated function, yet the species-specific dynamics of its distribution highlight the role of polyploidization as a powerful driver of satellite sequence reorganization. In the case of *D. villosum*, the detection of pericentromeric sequences in telomeric regions might be attributed to their importance in fundamental cellular processes such as homologous chromosome recognition, ensuring their proper segregation in meiosis and mitosis, and maintaining chromosomal structural integrity [86].

Thus, comparative cytogenetic study of satellite repeats allows us to conclude that the diploid species *D. villosum* and tetraploid *D. breviaristatum* represent genetically distinct but evolutionarily closely related species, each characterized by the unique patterns of satellite DNA chromosomal organization. The obtained cytogenetic data support allopolyploid origin of *D. breviaristatum*, resulting from the hybridization of *D. villosum* with another, possibly yet unknown or currently extinct, diploid species. This conclusion is consistent with previously published molecular genetic and phylogenetic studies, where species of the genus *Dasypyrum* form a common cluster while demonstrating clear species differentiation [5,9].

The chromosomal markers developed in this study, based on satellite repeats, open new avenues for investigating genomic architecture and evolutionary relationships within the genus *Dasypyrum*, as well as for identifying its chromatin in distant hybrids.

Despite the robustness of the obtained chromosomal patterns and the clear differentiation between *D. villosum* and *D. breviaristatum*, several limitations of the present study should be acknowledged. First, the analysis was conducted on single accessions per species, which does not fully capture the intraspecific variability of satellite DNA and may limit the generalization of our conclusions. Second, although FISH provides high-resolution visualization of repeat distribution, the relatively low sequencing coverage used for repeat identification may have resulted in incomplete detection of low-copy or highly diverged satellite families. These constraints highlight the need for expanded population-level sampling, deeper sequencing, and integrative quantitative cytogenomic approaches in future research.

## 4. Materials and Methods

### 4.1. Plant Materials

The following plant material was used in the study: the annual species *D. villosum* W6 21,717 (2n = 14, genome V), originating from Ukraine, and the perennial species *D. breviaristatum* PI 516,547 (2n = 28, genome VV^b^), originating from Morocco. All accessions were kindly provided by the USDA-ARS Germplasm Resources Information Network (GRIN).

### 4.2. Sequencing and Bioinformatics Analysis

Genomic DNA of *D. breviaristatum* was extracted from young leaves using the CTAB method [87]. The concentration of the extracted DNA was assessed using Qubit 4 (“Thermo Fisher Scientific”, Waltham, MA, USA), and quality assessment was performed by electrophoresis in a 1.5% agarose gel. Whole-genome sequencing was carried out on the Illumina NextSeq platform (“Illumina, Inc.”, San Diego, CA, USA) using a NextSeq 500/550 Mid Output Kit v2.5 (“Illumina, Inc.”, San Diego, CA, USA) as described in Illumina protocols for pair-end reads. Methodological details for the bioinformatics analysis pipeline used in this study are described in detail in Supplementary Materials File S1.

The primer sequences for the identified satellite repeat monomers are provided in Table 1.

Additionally, two satellite repeats were used as oligoprobes: CL169 TAMRA (5′-AAGCCTCCCTGACGCCCTCGAGCCAAAGACCGCA-3′) and CL173 FAM (5′-AATTTTGGG TCCCGGGGCGATCC-3′).

### 4.3. Fluorescence In Situ Hybridization (FISH)

Root tips from the studied *Dasypyrum* accessions were fixed and cytological preparations from root meristems were made according to the method described in [88]. The localization of satellite repeats on chromosomes of *D. villosum* and *D. breviaristatum* was performed using the fluorescence in situ hybridization (FISH) method according to the protocol from [89]. For detection, streptavidin-Cy3 ("Vector Laboratories", Peterborough, UK) and anti-digoxigenin-FITC (“Roche”, Mannheim, Germany) were used. After hybridization, chromosomes were counterstained with DAPI in Vectashield mounting medium (“Vector Laboratories”, Peterborough, UK). Subsequent re-hybridization with "standard" DNA probes (pSc119.2, pAs1, pTa-535, and (GAA)_10_) was performed for precise chromosome identification according to other studies [68,76,90,91,92]. For *D. villosum*, a combination of pSc119.2, pAs1, and (GAA)_10_ was used, while for *D. breviaristatum*, pSc119.2, pTa535, and (GAA)_10_ probes were applied. Signals were captured using a DFC 9000 GTC fluorescence microscope (“Leica”, Wetzlar, Germany) and processed in Adobe Photoshop 2017.1.1 (“Adobe Inc.”, San Jose, CA, USA).

## 5. Conclusions

This study presents an analysis of satellite repeats in *D. villosum* and *D. breviaristatum*, identified through whole-genome sequencing. Bioinformatics analysis revealed 14 satellite repeats in the *D. breviaristatum* genome, most showing homology with known repetitive sequences from the Triticeae tribe. FISH analysis established unique chromosomal localization patterns, classifying the repeats into three groups: predominantly terminal (CL100, CL110, CL134, CL135, CL147, CL165, CL169, CL173, and CL197), pericentromeric (CL127 and CL133), and mixed localization (CL9). Significant species-specific differences were revealed between diploid *D. villosum* and tetraploid *D. breviaristatum*, including tetraploid-specific repeats (CL110, CL134, CL135, CL147, CL165, and CL173) and reorganization of conserved sequence distribution (CL95). These results support the allopolyploid origin of *D. breviaristatum*. The identified satellite repeats can serve as molecular genetic markers for evolutionary, phylogenetic, and population studies in cereals, and for assessing chromosomal structure in cultivated cereal hybrids involving *Dasypyrum* species. We acknowledge the limitations of using single accessions and moderate sequencing coverage, which may not capture the full intraspecific variability and complexity of the repetitive genome. Future research should focus on expanded population-level sampling, deeper sequencing, and integrative cytogenomic approaches to fully elucidate the evolutionary dynamics of these repeats. The identified markers hold significant biotechnological potential for the efficient development and characterization of wheat–*Dasypyrum* introgression lines, facilitating the transfer of valuable agronomic traits.

## Figures and Tables

**Table 1:** Primer sequences for the satellite repeats.

Repeat	Primers
CL9	F: 5′-TTCGGACGAAAACCCGTCTG-3′
	R: 5′-GACGTGGCTTTGAATGGTGC-3′
CL95	F: 5′-GCGTTGGAAAGCTATTGGGG-3′
	R: 5′-GCCGGCTCATCGAAATTTGA-3′
CL100	F: 5′-GCCCGTTTCGTGGACTATTAC-3′
	R: 5′-AAACTGCGAGTGTTGATGACC-3′
CL110	F: 5′-AGAGCCTCCTCCTTCTATGC-3′
	R: 5′-CGGCTTCGGTAACATTTTGG-3′
CL127	F: 5′-ATGAGTAGACGCGTAGTGCG-3′
	R: 5′-CGGGGTGGTGTCGAAAATTG-3′
CL133	F: 5′-GGACACACACCCCTCACAAA-3′
	R: 5′-TGAAGGCCGTAAGAGGAAGC-3′
CL134	F: 5′-TCCGGGAAATCCCATTTGGC-3′
	R: 5′-ATGCCCTTTGGTTCATGGCT-3′
CL135	F: 5′-CCAACGAATCCTAAACCGCC-3′
	R: 5′-ACATGGATGGACACAATAGGGT-3′
CL147	F: 5′-CGACTGGAGCGGTTTAGGAA-3′
	R: 5′-CTTTCTCGGGGTTTGTGTGC-3′
CL153	F: 5′-ATGCTTGGCAAACAACCTTTAGC-3′
	R: 5′-CCTAGACTTGGTCTGTCATCT-3′
CL165	F: 5′-TACCCTTCGTCCCCTGTTGA-3′
	R: 5′-AATTGACACGTCGCTGGACT-3′
CL197	F: 5′-CATGAGTTGACCGCGAAGC-3′
	R: 5′-ATGATTTCCGTACAGCGGCG-3′

## Data Availability

The original contributions presented in this study are included in the article/Supplementary Material. Further inquiries can be directed to the corresponding author.

## Supplementary Materials

The following supporting information can be downloaded at: https://www.mdpi.com/article/10.3390/plants14243819/s1, Table S1: Repeatome annotation table for *D. breviaristatum* single RepeatExplorer2 assembly; File S1: Bioinformatic methods for comparative analysis of satellite DNA in *Dasypyrum* species; Figure S1: The karyotype of 14 *D. villosum* chromosomes with the studied satellite repeats is shown. The combinations of satellite repeats and oligo probes are shown on the left; the color of the probes corresponds to the color of the signal; Figure S2: The karyotype of 28 *D. breviaristatum* chromosomes with the studied satellite repeats is shown. The combinations of satellite repeats and oligo probes are shown on the left; the color of the probes corresponds to the color of the signal. References [93,94] are cited in the Supplementary Materials.
