# Supplementary material for: Comparative Analysis of Satellite DNA in Dasypyrum Species: Identification of Chromosomal Markers for V and Vb Subgenomes
Source: Plants (Basel). 2025 Dec 15;14(24):3819. doi: 10.3390/plants14243819 (PMC12737168; doi:10.3390/plants14243819)
Supplement: Supplementary file 1 [file plants-14-03819-s001.zip › Figures S1 and S2.pdf]

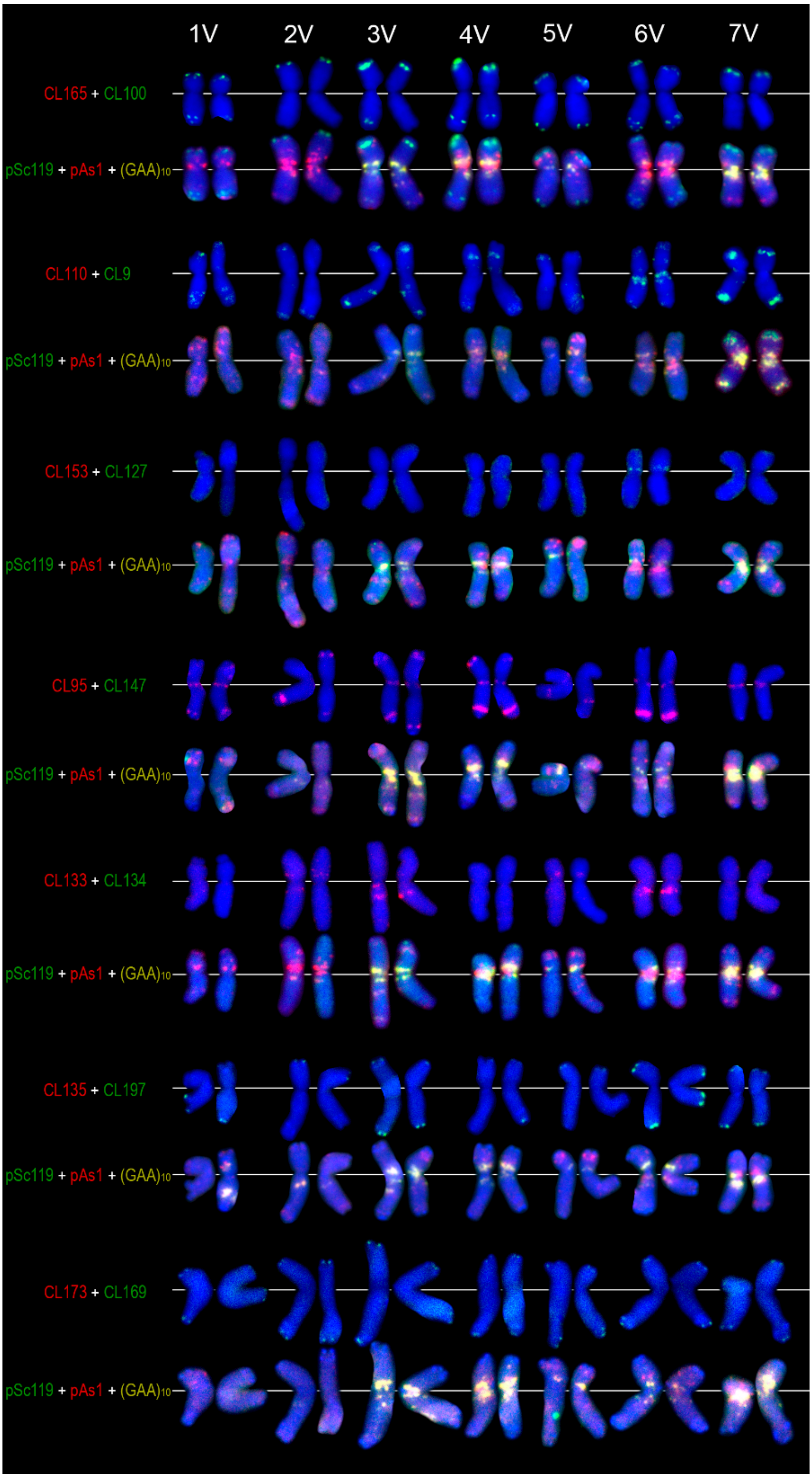


**Figure S1.** The karyotype of 14 *D. villosum* chromosomes with the studied satellite repeats is shown. The combinations of satellite repeats and oligo probes are shown on the left; the color of the probes corresponds to the color of the signal.

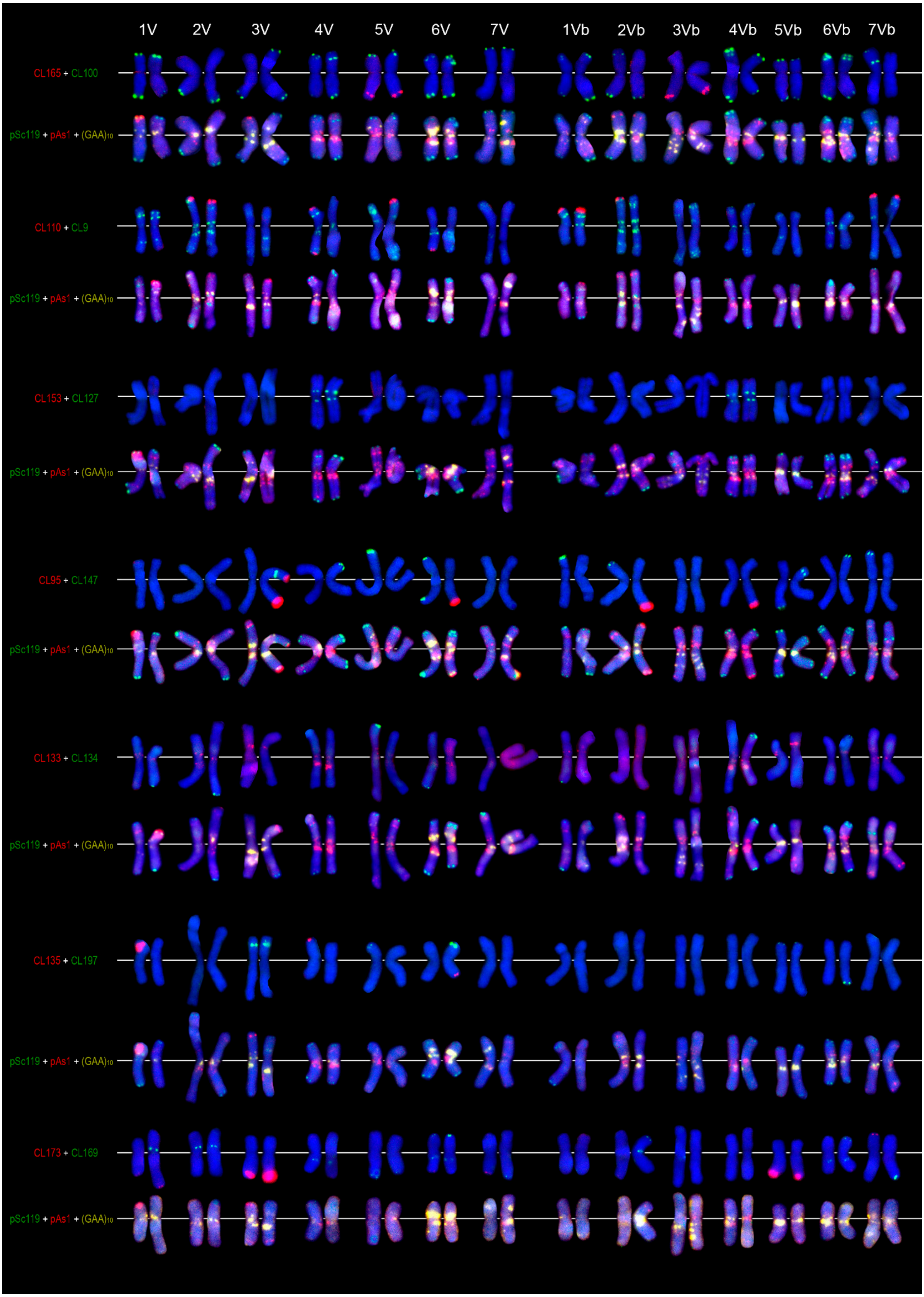


**Figure S2.** The karyotype of 28 *D. breviaristatum* chromosomes with the studied satellite repeats is shown. The combinations of satellite repeats and oligo probes are shown on the left; the color of the probes corresponds to the color of the signal.
