# Supplementary material for: Comparative Analysis of Satellite DNA in Dasypyrum Species: Identification of Chromosomal Markers for V and Vb Subgenomes
Source: Plants (Basel). 2025 Dec 15;14(24):3819. doi: 10.3390/plants14243819 (PMC12737168; doi:10.3390/plants14243819)
Supplement: Supplementary file 1 [file plants-14-03819-s001.zip › File S1.pdf]

Supplementary File S1. Bioinformatic Methods for Comparative Analysis of Satellite DNA in *Dasypyrum* Species: Identification of Chromosomal Markers for V and V^b Subgenomes

Contents

Introduction........2
Read Processing and Subsampling........2
RepeatExplorer/TAREAN Clustering Analysis........2
TAREAN Graph Visualization and Interpretation........2
Abundance Classification Criteria........3
NCBI Database Collection and Query Construction........3
BLAST Alignment Methodology........3
Software Versions and Implementation........5
References........5

Introduction

This document provides methodological details for the bioinformatics analysis pipeline used in this study. The pipeline integrates RepeatExplorer/TAREAN clustering with post-processing analysis and NCBI database alignment for repeat element identification, classification, and annotation.

Read Processing and Subsampling

Raw paired-end reads underwent quality control using FastQC (Andrews, 2010) and adapter removal using BBDuk (BBMap suite; Bushnell, 2014) with parameters ktrim=r, k=20, mink=10, hdist=2 (28 threads). Reads were randomly subsampled to 2 million read pairs (4 million reads) per sample using prepareReadsRE.py. This subsampling balances computational efficiency with representative repeat coverage, as RepeatExplorer algorithms maintain sensitivity for both high- and low-abundance repeats with subsampled datasets [93]. Subsampling used a deterministic random seed (PYTHONHASHSEED=0) to ensure reproducibility.

RepeatExplorer/TAREAN Clustering Analysis

Repeat element identification was performed using RepeatExplorer2 [93] and TAREAN [94] via seqclust from the repex_tarean repository (https://github.com/kavonrtep/repex_tarean). Analysis parameters: assembly_min=2 (mini- mum reads for contig assembly), mincl=0.0001 (minimum cluster size as proportion of total reads), r_value calculated as 80% of system memory in KB (180GB × 0.8 × 1024^2 ≈ 151000000 KB), threads=28. The mincl=0.0001 threshold enables detection of repeats at frequencies ≥0.01% of reads. The assembly_min=2 parameter allows consensus assembly from small clusters, ensuring rare repeat families are included.

TAREAN Graph Visualization and Interpretation

TAREAN employs graph-based visualization where nodes represent sequence reads and edges connect reads with significant sequence similarity. The layout algorithm minimizes edge crossings and high- lights structural patterns characteristic of different repeat types. Satellite DNA repeats form circular or ring-like structures reflecting tandem head-to-tail organization. Transposable elements (LTR retrotransposons, LINEs, SINEs) exhibit linear or branching patterns corresponding to dispersed genomic distribution. The connected component index (C) quantifies the proportion of nodes in the largest strongly connected component, measuring sequence homogeneity. C values >0.8 indicate highly conserved tandem repeats; lower values suggest

divergent or composite families. Graph layout images (graph_layout.png) are generated for each cluster and aid manual classification, particularly for distinguishing high-confidence satellites (circular topologies) from low-confidence candidates (fragmented patterns).

Abundance Classification Criteria

Repeat elements were classified using TAREAN's integrated system combining cluster proportion, adjusted proportion, satellite probability scores, and graph topology. High-confidence satellites (TAREAN rank 1) require: (1) satellite probability >0.7 (empirical estimates from manually annotated clusters; [94]), (2) connected component index C >0.8, (3) circular graph topology, and (4) cluster proportion >0.1% of total reads. Low-confidence satellites (rank 2) have satellite probability 0.3-0.7, C 0.5-0.8, and less regular topologies, requiring additional validation. Medium abundance clusters represent 0.05-0.5% of reads; high abundance >0.5%. The adjusted proportion metric corrects for partial removal of high-abundance satellites from all-to-all comparison during clustering, providing genomic proportion estimates that may differ from raw cluster proportions.

NCBI Database Collection and Query Construction

Reference sequences were collected from NCBI nucleotide database using taxonomic group-specific queries. For Triticeae (taxonomy ID 147389), the query was:

```
"triticeae"[porgn:_txid147389] AND ("repeat"[Title] OR "repetitive"[Title]
OR "enriched"[Title] OR "satellite"[Title] OR "transposon"[Title] OR
"gypsy"[ Title] OR "telomer"[Title] OR "marker"[Title] OR "anchor"[Title]
OR " retrotransposon"[Title] OR "microsatellite"[Title] OR "repeated"[
Title])
```

Sequences were filtered by: GC content 20-80%, ambiguous bases (N, X) <10% of length, sequence length 100-50,000 bp, and <50 annotated features (excluding over-annotated assemblies). Filtered sequences were compiled into FASTA format and converted to BLAST databases using makeblastdb (BLAST+ 2.16.0+). Metadata (accession numbers, lengths, GC content, source organisms) were preserved in CSV format.

BLAST Alignment Methodology

Query Sequences

TAREAN consensus sequences from all cluster ranks served as BLAST queries: rank 1 (high-

confidence satellites), rank 2 (low-confidence satellites), rank 3 (putative LTR retrotransposons), and rank 4 (ribosomal DNA). Consensus sequences represent the most probable repeat monomer sequences from k-mer-based analysis. For tandem repeats, both standard and x3 variants (three tandem copies) were generated to improve alignment sensitivity.

Alignment Algorithms

Three BLASTN task variants were used: megablast for high-homology alignments (>95% identity), dc-megablast for moderate-homology (70-95% identity), and blastn for general homology searches. This approach enables detection of both closely related and divergent repeat families.

Alignment Parameters

BLAST alignments used E-value threshold 0.1 for initial filtering (0.001 for weak/partial alignments, see below), minimum percent identity 60%, and default word size and gap penalties per task type. Results were formatted as tabular output (outfmt 6) including query/subject identifiers, percent identity, alignment length, mismatches/gaps, coordinates (qstart, qend, sstart, send), E-value, and bit score.

HSP Processing and Coverage Calculation

BLAST returns High-Scoring Pairs (HSPs), each representing a local alignment with associated coordinates. For each query-subject pair, all HSPs were processed collectively to calculate cumulative query coverage. Query coordinates (qstart, qend) from all HSPs were extracted as interval pairs. Intervals were sorted by start position and merged when overlapping or adjacent (within 1 bp): if current start ≤ previous end + 1, intervals were merged by extending to the maximum end position, preventing double-counting. Query length (qlength) was extracted from sequence names via pattern matching (e.g., "_x_568nt" → 568 bp). For x3 variant databases, query length was multiplied by 3. Total coverage = sum of merged interval lengths (end - start + 1). Coverage ratio = total_coverage / qlength.

Coverage Classification

Coverage ratios were classified into four categories: near-full (≥95%), where merged HSPs cover nearly the entire query (complete repeat matches); composite (≥80%), where multiple HSPs cover most of the query (composite matches or highly similar families); partial (≥60%), significant but incomplete coverage (partial matches, divergent repeats, or repetitive regions); weak (<60%), limited coverage (short motifs, partial homology, or spurious matches).

Filtering and Hit Selection

Weak and partial alignments (coverage <80%) required stricter E-value filtering (≤0.001) to reduce false positives from short repetitive matches. For each query-subject-task-database combination, all HSPs were considered together for coverage calculation, ensuring fragmented alignments spanning the same query region were properly merged. When multiple HSPs existed for a query-subject pair, the longest HSP was selected as representative after coverage calculation. Self-hits (identical query and subject) were removed. Levenshtein distance filtering (threshold 90%) was applied to NCBI results to remove redundant accessions with similar sequence names.

Database Sources

Alignments were performed against: (1) NCBI repeats database (curated sequences from NCBI as described above), (2) local reference databases (project-specific or manually curated sequences), (3) comparative databases (sequences from related species), and (4) important reference databases (experimentally validated or significant repeat families). Each database was available in standard and x3 (tandem repeat) variants.

Software Versions and Implementation

Software versions: RepeatExplorer2/TAREAN from repex_tarean repository (https://github.com/kavonrtep/repex_tarean), accessed via seqclust wrapper executing in conda environment (repeatexplorer) while maintaining compatibility with main pipeline environment (reportr); BLAST+ 2.16.0+ (bioconda); Python 3.7+ (reportr environment), Python 3.7.* (repeatexplorer environment); Snakemake ≥7.0. The RepOrtR pipeline (under development, not yet published) is implemented as a Snakemake workflow with modular rule definitions, enabling reproducible parallel execution and incremental processing to avoid redundant computation. RepOrtR is being developed to facilitate reproduction of repeat element analysis studies.

References

93. Novák, P.; Neumann, P.; Macas, J. Global Analysis of Repetitive DNA from Unassembled Sequence Reads Using RepeatExplorer2. Nature Protocols 2020, 15, 3745–3776, doi:10.1038/s41596-020-0400-y.

94. Novák, P.; Ávila Robledillo, L.; Koblížková, A.; Vrbová, I.; Neumann, P.; Macas, J. TAREAN: A Computational Tool for Identification and Characterization of Satellite DNA from Unassembled Short Reads. Nucleic acids research 2017, 45, e111–e111, doi:10.1093/nar/gkx257.
